# Neglected Populations Not to Be Forgotten: Tackling Antimicrobial Resistance in Neonatal Infections

**DOI:** 10.3390/antibiotics12121688

**Published:** 2023-12-01

**Authors:** Lucia Barcellini, Ilia Bresesti, Laura Folgori

**Affiliations:** 1 Department of Pediatrics, “V. Buzzi” Children’s Hospital, ASST FBF Sacco, 20154 Milan, Italy; lucia.barcellini@asst-fbf-sacco.it; 2 Neonatal Intensive Care Unit, “Filippo del Ponte” Hospital, ASST Settelaghi, 21100 Varese, Italy; ilia.bresesti@uninsubria.it; 3 Department of Medicine and Surgery, University of Insubria, 21100 Varese, Italy

## 1. Introduction

Making further progress in reducing child mortality hinges on lowering the annual count of neonatal deaths; currently, this stands at 2.5 million, 23% of which are the direct result of infections [1]. Diagnosing severe bacterial infections (SBIs) in neonates can be challenging, in both resource-poor and resource-rich settings, as the symptoms and signs are often non-specific and difficult to detect. Providing appropriate empirical treatment for SBIs is vital in reducing neonatal mortality.

The emergence of multidrug-resistant (MDR) pathogens poses a significant challenge to both high-income and low/middle-income countries (LMICs) [2]. First, neonates admitted to Neonatal Intensive Care Units (NICUs), especially premature infants, are identified as being at high risk for the development and transmission of MDR pathogens [3]. A recent report estimated that antimicrobial resistance might be responsible for approximately 30% of all global neonatal deaths due to sepsis [4]. A thorough understanding of local resistance patterns is necessary when selecting an appropriate empirical treatment for MDR infections in paediatric patients. Resistance profiles can vary significantly from region to region, making it crucial to tailor treatment strategies to local populations based on specific data regarding resistance in this group. Ignoring local resistance patterns and the misuse of antibiotics may lead to inappropriate treatment choices which can perpetuate resistance [3]. However, limited data is available regarding the impact of resistance profiles, virulence factors, the suitability of empirical treatment, and clinical characteristics on patient mortality. Second, the emergence of MDR pathogens in newborns is especially alarming due to the scarcity of effective therapeutic options for treating such infections. The proliferation of resistance to multiple classes of antibiotics has severely restricted the range of antibiotics that can be used to combat these infections. As a result, physicians often find themselves with few, if any, therapeutic choices when confronted with MDR infections in children and adults. Due to a depletion in the effectiveness of conventional antibiotics, older drugs that were previously not favoured for use in paediatrics are now being repurposed. This re-evaluation has been necessary to address the urgent need for treatment options. However, the use of older antibiotics without comprehensive data regarding their efficacy and safety in children carries inherent risks and uncertainties. Finally, Randomized Clinical Trials (RCTs) are the cornerstone of evidence-based medicine, making a significant contribution to advancing medical knowledge and patient care. However, when it comes to paediatric populations, conducting RCTs faces numerous challenges, resulting in a limited number of trials involving children. Ethical considerations represent a major hurdle in conducting CTs involving neonatal subjects. Concerns for the well-being and safety of children have led to stricter ethical standards and regulatory requirements within paediatric clinical research. Balancing the need for scientific knowledge with the ethical imperatives of minimising risks to vulnerable populations requires meticulous scrutiny and adherence to stringent ethical guidelines, often causing delays and complexities in trial initiation and execution. Enrolling an adequate number of neonates poses a significant challenge to conducting CTs in this group. Paediatric populations are inherently diverse, comprising various age groups, developmental stages, and health conditions. Finding an adequate number of eligible participants within these subsets can be daunting. The need for parental or guardian consent further complicates recruitment, requiring time, effort, and resources for the establishment of trust and securing informed consent. It is estimated that more than 50% of drugs used in paediatric patients are employed off-label, primarily due to the limited availability of age-specific data [5]. While this practice may provide treatment options, it raises questions about both safety and efficacy, as children and neonates may respond differently to medications compared to adults. Out of the forty antibiotics that have been approved for use in adults since 2000, only four of them have included dosing guidelines for neonates in their official labelling [6]. Currently, there are forty-three ongoing clinical trials for adult antibiotic treatments, while only six trials are actively enrolling neonatal patients [7].

With this Special Issue we aimed to foster international research to deepen the understanding of the role of the main determinants leading to adverse outcomes in newborns with SBIs within the scientific community. Furthermore, improving the surveillance programmes that collect neonatal AMR data and information regarding clinical outcomes is critical to allow us to create a benchmark for and design properly targeted interventions to decrease mortality. This Special Issue contains seven articles and five reviews, which will be described briefly in the following section.

## 2. Overview of Published Papers

Antimicrobial stewardship programmes represent diverse interventions designed to optimise antibiotic therapy. The primary goals of these programs are to enhance treatment efficacy, reduce medication-related side effects and infections (e.g., *Clostridioides difficile*), mitigate antimicrobial resistance, and manage costs. Importantly, these objectives are pursued without compromising patient outcomes, ensuring the maximal clinical effectiveness of antibiotics, minimising toxicity, and eliminating unnecessary financial burdens on patients and healthcare systems. Based on these principles, the review by Rallis D et al. (contribution 1) provides concise and up to date information on when to initiate antimicrobial therapy in neonates with suspected SBIs, presents current guidelines for choosing empirical antibiotics and the duration of treatment, and outlines the criteria for early discontinuation.

The use of colonisation screening as a predictor of infections caused by MDR bacteria in neonates has been implemented in several countries with the aim of detecting nosocomial infections in their early stages and to provide guidance for empirical treatment. However, as is shown in the paper from Bär A et al. (contribution 2), this technique has shown limited clinical significance due to its low positive predictive value. On the contrary, its high negative predictive value implies that achieving a negative result in colonisation screening avoids the necessity of using last-resort antibiotics and is a useful tool within hygiene surveillance.

The paper by Morreale C et al. (contribution 3) underlines the fact that the inappropriate use of antibiotics, particularly during the perinatal phase, is linked to enduring adverse outcomes, including the proliferation of antibiotic resistance and disruptions in the structure and functionality of the gut microbiota, which ultimately harm human health. In particular, they show that the swift interruption of antibiotic treatment (less than 72 h) and the use of probiotics may allow the gut microbiota composition to recover more quickly. This interesting review consolidates recent findings concerning the impact of antibiotic therapy on the neonatal gut microbiota and the resultant detrimental consequences on infant health. Furthermore, some potential strategies based on microbiome-related approaches to restoring a healthy microbiota in neonates have been explored. 

The neonatal early-onset sepsis (EOS) calculator has recently been introduced as a new strategy to manage infants at risk of sepsis and has shown promising but conflicting results. In the study carried out by Barbini MC et al. (contribution 4), the EOS calculator appeared to decrease the need for investigations in 1000 neonates ≥34 weeks of gestation. However, it did not lead to a reduction in the use of antibiotic therapy, highlighting that further research is still necessary to enhance its effectiveness. The study by Cavigioli F et al. (contribution 5) reports the results of a single-centre prospective study involving more than 3000 neonates between 2016 and 2020 in which three different workup algorithms were compared: the first approach relied on categorical risk assessment, the second employed a Serial Physical Examination (SPE) strategy for infants with EOS risk factors, and the third was linked to the EOS calculator, extending the universality of the SPE strategy. The unified approach demonstrated a substantial decrease in the utilisation of laboratory tests and antibiotic treatments in term and near-term newborns, leading to stable rates of EOS and mortality throughout the study period.

In her review, Dr Nusman CM (contribution 6) explores the potential of innovative targeted preventive and diagnostic methods for treating EOS from three distinct perspectives: the maternal (encouraging strategies that include *Group B Streptococcus* (GBS) prevention, research into GBS virulence factors, maternal immunisation, and antepartum biomarkers), the umbilical cord (with promising diagnostic methods), and the newborn (in the form of new biomarkers, novel microbiological techniques, clinical prediction, and monitoring strategies). The author concludes that an agreement on the definition of EOS, as well as the standardisation of research into innovative diagnostic biomarkers for future implementation, is urgently needed. 

MDR infections in the neonatal population represent a major concern. Several studies have been published reporting outbreaks of MDR infections in the context of NICUs. In this Special Issue, epidemic outbreaks caused by both gram-positives (contribution 7) and gram-negatives (contributions 8, 9 and 10) MDR bacteria have been published, with a particular focus on Extended-Spectrum Beta-Lactamase (ESBL) and Carbapenemase-producing *Klebsiella pneumoniae*. In all of these studies, the timely identification of the beginning of an event and the implementation of the infection control measures used have proven to be effective and have prevented the further spread of the bacteria within the ward.

Information concerning the management of gram-negative MDR bacteria in neonates is poor and primarily focused on older antimicrobial agents. This issue has been effectively summarised in the systematic review by Chiusaroli L et al. (contribution 11) underlying the fact that, although newer medications show promise, their cost prohibits their use in many LMICs. As a result, global strategies cannot be universally applied and will instead depend on the specific epidemiological conditions and available resources within each country. To conduct high quality studies and provide valuable insights for future trials, it is vital that we understand the obstacles faced when overseeing international multi-centre research studies and pinpoint feasible solutions for these contexts. The paper by Riddell A (contribution 12) offers a comprehensive examination of the challenges confronted by varied research teams across numerous countries and WHO regions in the context of the NeoOBS study. This study successfully gathered comprehensive, high-quality, long-term clinical and microbiological data from 19 hospitals in 11 countries worldwide. The challenges faced and the solutions implemented within this study can serve as valuable insights that can be used to shape strategies for forthcoming neonatal CTs.

## 3. Discussion and Conclusions

Addressing neonatal infections and antimicrobial resistance involves several crucial steps. 

A primary hurdle in treating neonatal sepsis lies in establishing a universally applicable definition across diverse settings and resource levels. A recent systematic review highlighted the significant heterogeneity in the definition of neonatal sepsis within randomised-controlled trials (RCTs); 85% of the studies examined relied on microbiological culture for a definitive diagnosis [8]. The limited availability and unreliability of microbial cultures contribute to the widely varying rates of ‘culture-negative’ or ‘suspected’ sepsis reported in the literature, with estimates of culture-negative sepsis rates among newborns ranging between 46% and 56% [9,10]. Despite a 2016 review leading to the introduction of the Sepsis-3 definition [11], which primarily focuses on ‘inflammatory host responses with end-organ impairment’ instead of microbiological isolates in sepsis in adults and the paediatric population, a consensus on neonatal sepsis remains elusive. This lack of agreement poses a substantial challenge to further research, resulting in non-comparable studies and the limited generalizability of these studies to the actual neonatal sepsis population in real-world scenarios. To address this, the suggested next steps involve a Delphi process, engaging international stakeholders to formulate a consensus on the definition of neonatal sepsis [8]. However, any definition of sepsis that incorporates organ dysfunction must first define normal organ function in the vulnerable preterm population, presenting a formidable challenge [8]. 

Secondly, there is an urgent need for a surveillance system and cohorts dedicated to understanding the current aetiology of neonatal sepsis in diverse settings, enabling the adjustment of empirical treatment strategies. Neonatal sepsis treatment typically begins empirically, and, in situations where cultures are unavailable, empirical treatment becomes the standard of care. Despite a growing body of evidence indicating the rapidly evolving epidemiology of the agents that cause neonatal sepsis [2,12,13], there are currently no adaptations of this epidemiology guided by the pathogens which are prevalent in specific regions. Establishing surveillance cohorts is crucial for gaining insights into the current aetiology of neonatal sepsis across diverse settings and adapting empirical treatment strategies accordingly. To achieve this goal, comprehensive microbiological data encompassing maternal pathogens, neonatal isolates, and the hospital environment are essential. This approach allows for the reconstruction of a detailed picture of the current sources of infection in newborns and the transmission mechanisms involved, as the traditional paradigm of early and late-onset disease appears outdated [14].

Finally, while the lack of trials that can generate high-quality evidence about neonatal infection is well recognised, it must be noted that there is also a scarcity of observational studies regarding neonatal sepsis. Advocating for a multinational observational cohort of infected newborns is important for reasons beyond understanding the epidemiology of the disease. 

RCTs remain the gold standard for determining the clinical efficacy of a treatment/intervention. However, there is a growing recognition of the importance of observational data in many medical fields. While observational studies have both strengths and limitations, they can be used to complement RCTs. Observational cohorts offer distinct advantages, including reduced time and cost, suitability for studying rare exposures and long-term outcomes, and the ability to encompass larger sample sizes, leading to greater statistical power and more precise effect estimates than RCTs. Additionally, these studies yield results with enhanced external validity and generalizability, considering various participants, practices, and settings. Observational studies have the advantage of including representative and diverse patient populations, allowing for treatment effects to be explored across different factors. International programmatic cohorts for cancer patients, cardiovascular disease, HIV, and TB have already proven to be a valuable source of data that can be used to answer relevant research questions in real-world scenarios [15,16,17]. Moreover, recommendations advocating for the use of observational studies in informing healthcare decisions have been made, recognising the importance replacing RCTs with observational data when the latter is very low quality, and using observational data in conjunction with RCTs when there are issues such as indirectness, imprecision, and inconsistency involved in the RCT. Finally, observational data should be used in sequence with RCTs to examine long term outcomes [18,19]. 

Advocating for the use of observational studies in newborns is crucial for several reasons. Firstly, newborns are a diverse population with unique characteristics, such as gestational age, birth weight, comorbidities, and the medical devices used in this group. This complexity often requires a personalised approach, making large-scale observational cohorts an excellent source of knowledge that can inform healthcare decisions. Secondly, there is limited participation in CTs due to various factors such as the perceived burden and lack of personal benefit, concerns about the safety of babies, trust in healthcare providers, and access to trial centres. Moreover, fewer RCTs are conducted on neonates compared to other populations due to the lack of time, resources, and interest in a relatively small population. Although we should strive to overcome these obstacles and conduct more RCTs in neonates, observational studies may complement such efforts and overcome these barriers.

To fully capitalise on the potential of observational research, it is essential that we increase the rigour of research methods. The scientific community often shows mistrust towards such research due to limitations that are not inherent to the study design but often stem from imprecise causal questions, inadequate protocols, and threats to validity. Investments in high-quality data and appropriate analytic methods are crucial for producing evidence from observational data. High-quality cohorts have recently emerged, providing opportunities for methodological research and the application of modern analytical approaches to tackle specific issues related to different patient cohorts [20,21,22]. 

It is about time that we unlocked the power of large observational cohorts to understand the epidemiology of and morbidity resulting from neonatal sepsis and considered this population’s complexity, contributing to better-informed policy decisions and improve patient outcomes. 

## List of Contributions

1. Rallis, D.; Giapros, V.; Serbis, A.; Kosmeri, C.; Baltogianni, M. Fighting Antimicrobial Resistance in Neonatal Intensive Care Units: Rational Use of Antibiotics in Neonatal Sepsis. *Antibiotics* **2023**, *12*, 508. https://doi.org/10.3390/antibiotics12030508.
2. Bär, A.; Schmitt-Grohé, S.; Held, J.; Lubig, J.; Hanslik, G.; Fahlbusch, F.B.; Reutter, H.; Woelfle, J.; van der Donk, A.; Schleier, M.; et al. Evaluating the Use of Neonatal Colonization Screening for Empiric Antibiotic Therapy of Sepsis and Pneumonia. *Antibiotics* **2023**, *12*, 189. https://doi.org/10.3390/antibiotics12020189.
3. Morreale, C.; Giaroni, C.; Baj, A.; Folgori, L.; Barcellini, L.; Dhami, A.; Agosti, M.; Bresesti, I. Effects of Perinatal Antibiotic Exposure and Neonatal Gut Microbiota. *Antibiotics* **2023**, *12*, 258. https://doi.org/10.3390/antibiotics12020258.
4. Barbini, M.C.; Perniciaro, S.; Bresesti, I.; Folgori, L.; Barcellini, L.; Bossi, A.; Agosti, M. The Management of Neonates ≥34 Weeks’ Gestation at Risk of Early Onset Sepsis: A Pilot Study. *Antibiotics* **2023**, *12*, 1306. https://doi.org/10.3390/antibiotics12081306.
5. Cavigioli, F.; Viaroli, F.; Daniele, I.; Paroli, M.; Guglielmetti, L.; Esposito, E.; Cerritelli, F.; Zuccotti, G.; Lista, G. Neonatal Early Onset Sepsis (EOS) Calculator plus Universal Serial Physical Examination (SPE): A Prospective Two-Step Implementation of a Neonatal EOS Prevention Protocol for Reduction of Sepsis Workup and Antibiotic Treatment. *Antibiotics* **2022**, *11*, 1089. https://doi.org/10.3390/antibiotics11081089.
6. Nusman, C.M.; Snoek, L.; van Leeuwen, L.M.; Dierikx, T.H.; van der Weijden, B.M.; Achten, N.B.; Bijlsma, M.W.; Visser, D.H.; van Houten, M.A.; Bekker, V.; et al. Group B Streptococcus Early-Onset Disease: New Preventive and Diagnostic Tools to Decrease the Burden of Antibiotic Use. *Antibiotics* **2023**, *12*, 489. https://doi.org/10.3390/antibiotics12030489.
7. Nusman, C.M.; Blokhuis, C.; Pajkrt, D.; Visser, D.H. Staphylococcal Scalded Skin Syndrome in Neonates: Case Series and Overview of Outbreaks. *Antibiotics* **2023**, *12*, 38. https://doi.org/10.3390/antibiotics12010038.
8. Priante, E.; Minotti, C.; Contessa, C.; Boschetto, M.; Stano, P.; Dal Bello, F.; De Canale, E.; Lolli, E.; Baldo, V.; Baraldi, E.; et al. Successful Control of an Outbreak by Phenotypically Identified Extended-Spectrum Beta-Lactamase–Producing Klebsiella pneumoniae in a Neonatal Intensive Care Unit. *Antibiotics* **2022**, *11*, 1649. https://doi.org/10.3390/antibiotics11111649.
9. Pruss, A.; Kwiatkowski, P.; Masiuk, H.; Bilska, I.; Giedrys-Kalemba, S.; Dołęgowska, B. Epidemiological Analysis of Extended-Spectrum β-Lactamase-Producing Klebsiella pneumoniae Outbreak in a Neonatal Clinic in Poland. *Antibiotics* **2023**, *12*, 50. https://doi.org/10.3390/antibiotics12010050.
10. Agosta, M.; Bencardino, D.; Argentieri, M.; Pansani, L.; Sisto, A.; Ciofi Degli Atti, M.L.; D’Amore, C.; Bagolan, P.; Iacobelli, B.D.; Magnani, M.; et al. Clonal Spread of Hospital-Acquired NDM-1-Producing Klebsiella pneumoniae and Escherichia coli in an Italian Neonatal Surgery Unit: A Retrospective Study. *Antibiotics* **2023**, *12*, 642. https://doi.org/10.3390/antibiotics12040642.
11. Chiusaroli, L.; Liberati, C.; Caseti, M.; Rulli, L.; Barbieri, E.; Giaquinto, C.; Donà, D. Therapeutic Options and Outcomes for the Treatment of Neonates and Preterms with Gram-Negative Multidrug-Resistant Bacteria: A Systematic Review. *Antibiotics* **2022**, *11*, 1088. https://doi.org/10.3390/antibiotics11081088.
12. Riddell, A.; Cook, A.; Khavessian, N.; Ellis, S.; Bilardi, D.; Correia, E.; Kostyanev, T.; Nardone, A.; Russell, N.; et al. Challenges in the Implementation of the NeoOBS Study, a Global Pragmatic Observational Cohort Study, to Investigate the Aetiology and Management of Neonatal Sepsis in the Hospital Setting. *Antibiotics* **2023**, *12*, 923. https://doi.org/10.3390/antibiotics12050923.
